# Vulnerable robots positively shape human conversational dynamics in a human–robot team

**DOI:** 10.1073/pnas.1910402117

**Published:** 2020-03-09

**Authors:** Margaret L. Traeger, Sarah Strohkorb Sebo, Malte Jung, Brian Scassellati, Nicholas A. Christakis

**Affiliations:** ^a^Yale Institute for Network Science, Yale University, New Haven, CT 06520;; ^b^Department of Sociology, Yale University, New Haven, CT 06520;; ^c^Department of Computer Science, Yale University, New Haven, CT 06520;; ^d^Department of Information Science, Cornell University, Ithaca, NY 14853;; ^e^Department of Biomedical Engineering, Yale University, New Haven, CT 06520;; ^f^Department of Statistics and Data Science, Yale University, New Haven, CT 06520

**Keywords:** human–robot interaction, groups and teams, conversational dynamics

## Abstract

Social robots are becoming increasingly influential in shaping the behavior of humans with whom they interact. Here, we examine how the actions of a social robot can influence human-to-human communication, and not just robot–human communication, using groups of three humans and one robot playing 30 rounds of a collaborative game (*n* = 51 groups). We find that people in groups with a robot making vulnerable statements converse substantially more with each other, distribute their conversation somewhat more equally, and perceive their groups more positively compared to control groups with a robot that either makes neutral statements or no statements at the end of each round. Shifts in robot speech have the power not only to affect how people interact with robots, but also how people interact with each other, offering the prospect for modifying social interactions via the introduction of artificial agents into hybrid systems of humans and machines.

## Significance

Prior work has demonstrated that a robot’s social behavior has the ability to shape people’s trust toward, responses to, and impressions of a robot within human–robot interactions. However, when the context changes to interactions within a group involving one robot and multiple people, the influence of the robot on group behavior is less well understood. In this work, we explore how a social robot influences team engagement using an experimental design where a group of three humans and one robot plays a collaborative game. Our analysis shows that a robot’s social behavior influences the conversational dynamics between human members of the human–robot group, demonstrating the ability of a robot to significantly shape human–human interaction.

Human behavior is very sensitive to the influence of other people in their groups. Verbal behavior, in particular, plays a special role in establishing successful social interactions and cooperation (1). Additionally, successful social interactions are remarkably dependent on group dynamics, especially when contrasting a one-on-one (dyadic) interaction with an interaction occurring across several members of a group (2, 3). Social influence across social ties can enable the spread of phenomena as diverse as emotions, voting, and cooperation, in both natural and laboratory settings (4–7).

Just as humans are capable of influencing the behaviors of the people with whom they interact, robots may exert similar effects. For example, robots can alter how well human participants complete a task (8–12) and how humans respond to requests (13–15) in human–robot interactions. Studying such one-on-one interactions between humans and robots is important to understanding how robots can affect direct human–robot relationships. For example, algorithms programmed with simple communication capacities, such as “cheap talk,” are able to effectively improve one-on-one human–robot cooperation to a level on par with human–human cooperation in online games (16). However, analyzing interactions in groups with several humans and one robot, compared to one-on-one interactions, can illuminate other aspects of how robots influence human behavior between the humans themselves.

Research on group interactions is less common, but extant work has shed light on several novel aspects of human–robot interactions. For example, a social robot can shape the roles members of a group assume through gaze direction (17). Robots can also be programmed with algorithms that alter their behavior to emphasize different aspects of the group, such as increasing group cohesion, productivity, sense of peace, or engagement (18–21). Furthermore, in settings with many human participants, robots can guide interactions between humans by adjusting their speech, focus, and actions (9, 22–25). This effect is especially pronounced when the content of robot speech is based on group emotions, which can lead to a more positive view of the robot (26). As robots increasingly populate our homes and workplaces, the ubiquitous presence of such groups composed of humans and machines could alter how humans relate to both robots and each other (27–29).

For a group to sustain and achieve collective outcomes, it is extremely helpful if its members socially engage with one another and are willing to communicate. Groups without social interaction are less able to learn from each other and work together (30, 31). One way to establish and promote social engagement within groups is through vulnerable expressions. Vulnerability focuses individuals on others, which encourages interpersonal connection (32). In interpersonal interactions, vulnerability is often expressed as self-disclosure or personal stories (which increase solidarity) and humor (which alleviates tension) (33–36). But this can also be extended to nonhuman agents, like robots, by programming them to exhibit these behaviors (37, 38). For instance, when robots disclose emotions in a collaborative task, humans are more likely to project feelings of companionship onto the robot (37). Expressions of vulnerability by a robot have also been shown to positively influence interpersonal behaviors (e.g., laughing with team members and liking the robot more) within a human–robot group (39, 40).

While this prior work offers evidence of a robot’s capacity to influence how human group members interact in the presence of a robot, this work has not examined whether these changes are sustained over time or if the influence of robot utterances extends to the dynamics of the discussion between the humans themselves. Here, we focus on the ability of a robot to improve human discussion in a collaborative task. We test whether a robot programmed to utter vulnerable statements throughout a sustained group interaction can influence the conversational dynamics between human members of the group.

In this experiment, 153 participants were assigned to 51 groups consisting of 3 human participants and 1 robot. The groups worked collaboratively, with the social robot, over 30 consecutive rounds, to play our custom-built, tablet-based game (39). This game was developed so that the human members of the group and the robot were perceived to all be part of the same team (see *SI Appendix* for more information). A limited version of this paradigm has been shown to increase engagement with robots themselves as well as how likely human participants are to explain their mistake and console their teammates in the game (39). Groups were assigned to one of three conditions: the vulnerable condition, the neutral condition, or the silent condition. In the vulnerable condition, the robot made a vulnerable comment at the end of every round; in the neutral condition the robot made a task-related comment at the end of every round (see *SI Appendix* for more information); and in the silent condition the robot did not speak at the end of every round.

## Results

We used multilevel modeling to account for the clustered structure of the data (participants in groups) and, when needed, the longitudinal aspects of the data (rounds within participants within groups). Further details on the analysis can be found in *SI Appendix*.

### Total Talking Time.

**Fig. 1.**
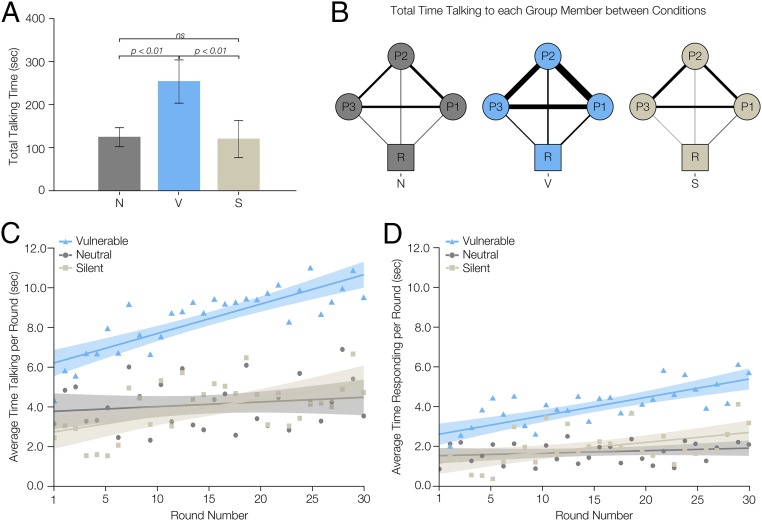
Total talking time by condition. Compared to the neutral and silent conditions, human participants in the vulnerable condition spoke more, in total, to the other participants in their group, and increasingly across game rounds. In *A*, we see that participants in the vulnerable condition (V) spoke significantly more than participants in either the neutral (N) or silent (S) condition (*n* = 153 participants). In *B*, the line widths represent the amount of talking by human participants toward their teammates who are connected by the line, in seconds (summed across all groups within a condition (*n* = 153 participants)). R = robot; P1, P2, and P3 = human participants, in their relative positions around the table. In *C* and *D*, the shaded area around each line represents a 95% confidence interval (*n* = 4,590 rounds); the dots represent the condition average for that round. In *C*, the vulnerable condition has more talking in every round, and the slope (i.e., the rate of increase in talking per round, across rounds) is higher than the neutral condition (but indistinguishable from the silent condition). In *D* we see that, compared to the neutral condition, those in the vulnerable condition respond more over time to their fellow human group members (*n* = 4,590 rounds).

There was a substantial difference between conditions in the total amount of time spent talking by participants, where those in the vulnerable condition spoke twice as much over the course of the game ($\bar{x_{V_{i}}}=253.60\;\text{s}$, SD = 184.41 s) compared to those in the neutral condition ($\bar{x_{N_{i}}}=\mathrm{124}\mathrm{.23}\;\text{s}$, SD = 78.78 s) and the silent condition ($\bar{x_{S_{i}}}=119.86\;\text{s}$, SD = 148.17 s). This difference was statistically significant between the vulnerable and neutral conditions (*c* = 140.68, *P* = 0.001) and the vulnerable and silent conditions (*c* = 124.52, *P* = 0.004), even after adjustment for age, extraversion, gender, and familiarity, using regression models, but there was no significant difference between the silent and neutral conditions (*c* = 16.15, *P* = 0.70) (see Fig. 1*A* and *SI Appendix*, Table S2).

In Fig. 1*B*, we show the total time participants spent talking to each of the other human participants and the robot, represented by the line width of the connections in the group network. The vulnerable robot condition enhanced interhuman conversation. In the neutral condition, across all groups, participants spoke to their human teammates for 83.22 min (4,993 s) and to the robot for 10.27 min (616 s) over the course of the game. In the vulnerable condition, participants spoke to their human teammates and the robot more than twice as much [178.38 min (10,703 s) and 24.02 min (1,441 s), respectively]. In the silent condition, participants spoke to their human teammates for similar amounts of time to the neutral condition [83.07 min (4,984 s)] but spoke to the robot very little [5.61 min (336 s)].

Additionally, those in the vulnerable condition spoke progressively more over time (across rounds in the game) ($\bar{x_{V_{i}}}=8\mathrm{.45}\;\text{s}$, SD = 8.33 s) compared to those in the neutral condition ($\bar{x_{N_{i}}}=\mathrm{4.14}\;\text{s}$, SD = 4.99 s) as demonstrated by the significant interaction effect between round and experimental condition with respect to the vulnerable and neutral conditions (*c* = 0.13, *P* = 0.03), although there is no significant difference between the silent condition ($\bar{x_{S_{i}}}=4\mathrm{.00}\;\text{s}$, SD = 6.73 s) and neutral condition (*c =* 0.06, *P* = 0.32) or the vulnerable and silent condition (*c* = 0.07, *P* = 0.27) (see Fig. 1*C* and *SI Appendix*, Table S3).

We further find that the difference in the amount of talking by those in the vulnerable condition was primarily driven by one type of utterance, namely, the communications between the human players themselves, with an increase in responses to other humans over time. In other words, participants in the vulnerable condition ($\bar{x_{V_{i}}}=3\mathrm{.99}\;\text{s}$, SD = 5.16 s) increased the amount of time they spent responding to the utterances of their other human group members as the game progressed (Fig. 1*D*) compared to those in the neutral condition ($\bar{x_{N_{i}}}=1\mathrm{.72}\;\text{s}$, SD = 2.76 s) (*c* = 0.08, *P* = 0.04), although there was no difference between the silent condition ($\bar{x_{S_{i}}}=1\mathrm{.96}\;\text{s}$, SD = 4.01 s) and the neutral condition (*c* = 0.04, *P* = 0.36) or the vulnerable condition and the silent condition (*c* = 0.04, *P* = 0.27), in terms of the increase over rounds. No other utterance category (see *SI Appendix* for details) was statistically significant across rounds of the game (*SI Appendix*, Table S4).

### Equality in Conversation.

**Fig. 2.**
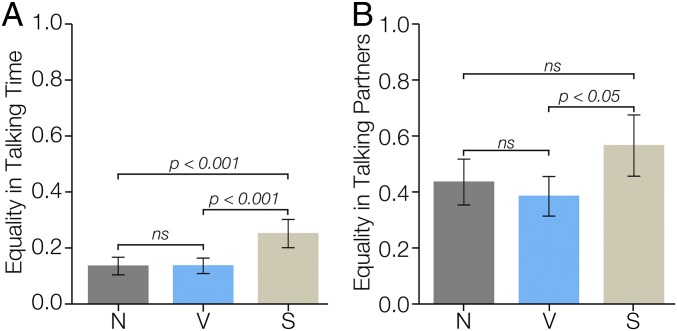
Equality of conversation by condition. Although there was (*A*) no statistical difference between the vulnerable (V) and neutral (N) conditions in the equality in talking time (*n* = 150 participants; one group did not speak at all and was excluded), the silent (S) condition was less equal than either of the other two conditions. (*B*) Also, participants in the vulnerable condition directed their utterances more equally to each of their human group members than participants in the silent condition, as measured by the total amount of time spent talking to each participant’s two human partners (*n* = 144 participants; participants who didn’t speak at all or who did not make directed utterances were excluded).

In addition to examining the amount of participants’ speech, we also explored how equally participants’ speech durations were within a group as well as how evenly participants distributed their speech to the two other human members in the group. To quantify the former, we used the following “equality in talking time” (*E_TT_*) metric:

$$E_{TT_{i}}=c\left|\frac{\tau_{i}}{\sum_{1}^{n}\tau_{i}}-\frac{1}{n}\right|,$$

where $\tau_{i}$ represents the total amount of time participant i spoke during the game, n is the number of human participants (3 in this case), $\sum_{1}^{n}\tau_{i}$ is the total amount of time participant i’s group spoke during the game, and c is a normalizing constant, causing $E_{TT_{i}}$ to have a range of [0, 1]. $E_{TT_{i}}$ takes on a value of 0 when a participant speaks for a third of the total amount of time their group speaks and a value of 1 when a participant speaks and their group members did not speak at all. We found that equality of time speaking did not differ between the vulnerable robot condition ($\bar{x_{E_{TT_{V_{i}}}}}=0\mathrm{.14}$, SD = 0.10) and the neutral robot condition ($\bar{x_{E_{TT_{N_{i}}}}}=0\mathrm{.14}$, SD = 0.11) (*c* = −0.03, *P* = 0.88, see *SI Appendix*, Table S5), but there is a significant difference between the neutral and silent conditions ($\bar{x_{E_{TT_{S_{i}}}}}=0\mathrm{.25}$, SD = 0.17) (*c* = −0.63, *P* = 0.001) and the vulnerable and silent conditions (*c* = −0.66, *P* = 0.0004). In other words, the distribution of speech by participants in the vulnerable condition did not differ from that in the neutral condition, but participants in the silent condition had the least equal distribution of talking time, as seen in Fig. 2*A*. Thus, the mere presence of a robot that communicates may enhance the equality of talking time in conversation among humans in a group.

To examine how evenly distributed each participant’s utterances were toward their fellow human teammates in the human–robot group, we created an “equality in talking partners” $\left(E_{TP}\right)$ metric as follows:

$$E_{TP_{i}}=\frac{\left|\tau_{\left(Pi,Pj\right)}-\tau_{\left(Pi,Pk\right)}\right|}{\tau_{\left(Pi,Pj\right)}+\tau_{\left(Pi,Pk\right)}},$$

where $\tau_{\left(Pi,Pj\right)}$ represents the talking time of participant i’s speech specifically directed at participant j during the game and $\tau_{\left(Pi,Pk\right)}$ represents the talking time of participant i’s speech specifically directed at participant k during the game. In other words, this measures how balanced a participant’s speech is toward the two other human members of their group over the whole game. If a participant directs all of their speech to one participant and none to the other, that participant gets a value of 1. If a participant speaks for the exact same amount of time to each of the other two participants, that participant will receive a value of 0. In other words, values of 1 represent perfect inequality and 0 represents perfect equality. Every human–human pairwise comparison is made for each participant in each group. We found no evidence that participants in the vulnerable condition ($\bar{x_{E_{TP_{V_{i}}}}}=0.38$, SD = 0.26) distributed their speech more equally between their fellow human group members than those in the neutral condition ($\bar{x_{E_{TP_{N_{i}}}}}=0.43$, SD = 0.29) (*c* = −0.38, *P* = 0.16) or between the silent condition ($\bar{x_{E_{TP_{S_{i}}}}}=0.57$, SD = 0.34) and neutral condition (*c* = 0.36, *P* = 0.25). However, the vulnerable condition is significantly more equal than the silent condition (*c* = −0.74, *P* = 0.01), as shown in Fig. 2*B* (*SI Appendix*, Table S6). In other words, speech seems to be more balanced in the conditions with a vulnerable robot compared to a silent robot.

### Self-Reported Group Dynamics.

Using comments that the participants provided in the postexperiment survey, we analyzed how participants perceived their own group’s dynamics (*SI Appendix*, Table S7). Comments were reliably coded by two coders (see *SI Appendix* for details) into the following four categories: quiet, positive, supportive, and fun.

We found that those in the vulnerable condition thought of their groups as being less quiet than did those in the neutral condition (*P* = 0.03) and that the silent condition did not differ from the neutral condition (*P* = 0.29)—in accordance with the foregoing analyses of actual talk time. The vulnerable condition did not differ from the silent condition (*P* = 0.23). Those in the vulnerable condition viewed their groups as more positive than did those in the neutral condition (*P* = 0.04) and the silent condition (*P* = 0.01). There was no difference between the silent and neutral conditions (*P* = 0.54). Those in the vulnerable condition also viewed their groups as being more fun than did those in the neutral condition (*P* = 0.03) or the silent condition (*P* = 0.02), and the silent and neutral conditions did not differ (*P* = 0.71). There was no difference between the vulnerable and neutral conditions in how supportive participants found their groups (*P* = 0.77), nor was there a difference between the silent and neutral conditions (*P* = 0.13) or the vulnerable and silent conditions (*P* = 0.19). See *SI Appendix* for more details. In summary, participants in the vulnerable condition described their groups as more pleasant overall than those in either of the two other conditions.

## Discussion

We experimentally evaluated human-to-human conversational dynamics in a group with three human group members and either a vulnerable, neutral, or silent robot group member. Participants in a group with a vulnerable robot spoke more overall than participants in a group with a neutral or silent robot and increasingly over the rounds of the game than participants in a group with a neutral robot. This difference is largely driven by responses to other human group members, which demonstrates an increasing social engagement effect in the conversation. It is likely that the vulnerable statements made by the robot helped participants feel more comfortable conversing with their group members. In short, it is not just the presence of a speaking robot in a hybrid system that changes human–human communication, but rather the nature of the robot’s speech––specifically, speech that is vulnerable.

As the total amount of time spent talking may not fully capture the nature of the conversation, we also examined the equality of each member’s speech amount, relative to their group members, as well as how evenly distributed each member’s talking was between their fellow human group members. We did not find a significant difference in the equality in talking time measure in groups with the vulnerable robot and in groups with the neutral robot, but participants in the silent robot condition had significantly less equal talking times, as if the mere presence of a talking humanoid robot helped even out the total amount of speaking between each human. Additionally, we found that those in a group with the vulnerable robot distributed their talking more equally between the other two human members of their group than those in a group with the silent robot. In short, we found that the vulnerable utterances by the robot not only influenced groups to talk more, but also positively shaped the directionality of the utterances to be more evenly balanced between the other two human members of the group compared to the silent condition.

These results demonstrate that robots are able to positively shape group conversational dynamics and emphasize the influential role that robots can have on the way we converse and interact with each other in the context of human–robot groups. As robots and other forms of machine intelligence (such as digital assistants and online bots) become increasingly prevalent in our daily lives in what we have called “hybrid systems,” (9) they will likely shape our actions, relationships, and conversations. They will critically affect our interactions with other people. Without an understanding of how machines may influence our connections with the people around us, we may encounter negative and unexpected consequences of interacting with these robots (41, 42).

For instance, there has been a great deal of concern about the capacity for physical robots and autonomous bots to damage human interactions (for instance by causing car accidents or spreading propaganda), but equally it is the case that such agents may improve human welfare. Prior work has shown the capacity for online bots to facilitate coordination (9) or decrease racist speech online (43). Here, we show that physical robots can improve conversational dynamics among humans. We believe that research investigating the consequences of social agents on human behavior, like the work presented here, will help agent designers optimally take such social spillovers into account and may help inform the general public on how to use artificial intelligence (AI) technology in beneficial ways.

Machines endowed with particular kinds of programming might have effects not only by modifying the behavior of humans with whom they directly interact, but also by modifying the behavior between the humans themselves when they interact with other humans in a group, thus creating diverse sorts of behavioral cascades. This can happen even when people actually know they are interacting with machines, as in the present case [and as shown in other work (9)]. In this sense, even simple AI agents might be able to serve a beneficial function, shaping the actions of their human counterparts and modifying human–human interactions and not just human–robot interactions. Our work illustrates the idea that, in hybrid systems of humans and machines, robots could help people work better together.

## Materials and Methods

### Recruitment Procedures.

After approval by the Yale University Institutional Review Board (IRB), we recruited 195 participants, but due to experimental malfunctions (e.g., video/audio failing to record, system glitches that prevented game play), 153 participants (51 groups) are included in this analysis. Most of our participants (73%) had very low familiarity with the other participants in their group. Further details, along with the regression procedures used to control for variation in measured covariates across treatment groups, are provided in *SI Appendix*.

### The Collaborative Game.

To set up a collaborative environment where the robot’s vulnerability might help to ease group tension and facilitate positive group dynamics, we designed a collaborative game to be played on individual tablets where each human participant and an autonomous humanoid robot would be equal contributors (see *SI Appendix* for more details). This collaborative game for three humans and one robot (a Softbank Robotics NAO robot) was built to be played on individual Android tablets running our bespoke Railroad Route Construction game.

The game we developed was created to give players the sense that they are playing collaboratively—by making success contingent on everyone in the group completing their part of the game successfully for the whole group to succeed. In other words, if one person fails, everyone fails. To successfully complete a round, each player needs to place eight pieces along the most efficient route for the railroad. This same game procedure was completed for all 30 rounds for each group. The group’s score was the total number of successfully completed rounds for that group.

### Experimental Procedure.

When participants arrived, they were given a consent form (or adolescent assent form after receiving parental consent), which was signed by the participant (and guardian, if applicable) before participating in any aspect of the study. Then, the participants were given a preexperiment survey. After completion of the survey, the participants entered the experiment room to play the game described above. Further details are provided in *SI Appendix*. The 3 human participants and the robot played 30 rounds of the Railroad Route Construction game. In the neutral condition, the robot made neutral utterances at the end of each round and did not acknowledge when it had made a mistake. In the vulnerable condition, the robot made vulnerable utterances when each round finished, which included acknowledging its mistakes. In the silent condition, the robot did not say anything at the end of each round. In the vulnerable and neutral conditions, the end-of-round utterances were approximately the same length across conditions. For more details, consult *SI Appendix*.

During the game, each player would take a railroad track piece from the options on the right-hand side of the screen and drag it to the active play area to complete the track (see *SI Appendix* for more details). Each player was instructed to build the optimal route during each round by creating the shortest contiguous track on their tablet. The group succeeded in a round if each player completed their path. The group failed a round when at least one participant failed to complete their track.

Over the course of the game, by design, each participant, including the robot, failed twice (see *SI Appendix* for details). In the case of the humans, we engineered this outcome by making no suitable track parts available to be placed on the game-play surface. Participants did not perceive this as accidental and felt responsibility for their “failure.” After the game, participants completed a postexperiment survey. When this survey was complete, they were given a debrief form about the true nature of the game and a payment for their time.

Vulnerable utterances made by the robot after each round of the game in the vulnerable condition were constructed based on the principles of psychological safety (44). The utterances by the robot were of three types: self-disclosure, personal story, and humor. Both self-disclosure and telling a personal story show vulnerability through the sharing of personal information with another member of the group (34). The robot used self-disclosure in utterances such as: “Sorry guys, I made the mistake this round. I know it may be hard to believe, but robots make mistakes too.” Personal stories from the robot consisted of sharing information about prior experiences and passions. These utterances were statements such as: “Awesome! I bet we can get the highest score on the scoreboard, just like my soccer team went undefeated in the 2014 season!” Lastly, the robot expressed vulnerability by using humor, as humorous expressions constitute a social gamble that has the potential to reduce discomfort within the group (35, 36). An example of a humorous phrase the robot used was “Sometimes failure makes me angry, which reminds me of a joke: Why is the railroad angry? Because people are always crossing it!” (We quite realize that this joke is corny, but that was the point.) In contrast, neutral statements were fact-based and did not contain any personal information or humor. For example, the robot would say, “We have completed 17 rounds thus far and have successfully built 76 percent of them.” All end-of-round utterances can be found in *SI Appendix*.

To verify that the comments made by the robot at the end of each round were perceived to be vulnerable in the vulnerable condition and fact-based in the neutral condition, we asked 210 human judges (via Amazon Mechanical Turk) to assess pairs of utterances. Judges were provided with random pairs of utterances (one utterance from each condition in a pair) and were asked which of the two indicated more vulnerability. By a ratio of nearly 3:1, the judges selected the utterances that we designed to be vulnerable as vulnerable (details can be found in *SI Appendix*).

### Measures of Conversational Dynamics.

In order to analyze the conversational dynamics within groups, we transcribed and categorized each utterance made by the participants using ELAN software (45). All utterances made throughout the game were included. These utterances fell broadly into two categories: comments and responses. We define comments as utterances that are addressed to others within the group, but that are not contingent on what has been said previously in the conversation. In other words, comments are new thoughts. In contrast, we define a response as an utterance that is dependent on what has just been said in the conversation. Often, responses are to comments, but they can also be a response to a response. Both comments and responses could be directed speech to certain individuals in particular or to the group as a whole (see *SI Appendix* for details on the utterance categorizations). For example, a comment would be an utterance such as, “Alright, we need to beat the top team” followed by a response of “We can do it!” The coder agreement (Cohen’s κ) across the four coders on these categorizations was *k* = 0.92.

### Control Variables.

Because characteristics of the participants could shape their willingness to engage socially with their group members, we asked participants to report their age, gender, familiarity with others in their group, and degree of extraversion.

Before entering the experiment room, we provided participants with a survey in which we asked them to select their familiarity with the two other human participants in their group on a 5-point scale. In addition, we asked participants if they were “Facebook friends” with, or had the telephone numbers of, the other members of their group. For more details on how familiarity was calculated, consult *SI Appendix*. In the postexperiment survey, we asked participants about the psychological safety of the group, what the participant thought of the robot, how extraverted the participant was, and a series of general questions about the game and the group.

We believed that whether or not a participant was extraverted would have an impact on their willingness to engage socially with their other group members. To avoid priming participants, we asked participants 6 yes or no questions from the abbreviated, revised Eysenck personality questionnaire (EPQR-A) (46) in the postexperiment survey. Participants were given a score from 0 (lowest extraversion) to 6 (highest extraversion) by adding the number of affirmative answers to the 6 questions in our survey. More details can be found in *SI Appendix*.

### Data Availability.

Data and reproducible R scripts are available on the Human Nature Lab website at http://humannaturelab.net/publications/vulnerable-robots (raw participant videos are not available due to IRB stipulations).

## Supplementary Material

Supplementary File
