# Supplementary material for: Vulnerable robots positively shape human conversational dynamics in a human–robot team
Source: Proc Natl Acad Sci U S A. 2020 Mar 9;117(12):6370–5. doi: 10.1073/pnas.1910402117 (PMC7104178; doi:10.1073/pnas.1910402117)
Supplement: Supplementary File [file pnas.1910402117.sapp.pdf]

PNAS
www.pnas.org

Supplementary Information for

Vulnerable Robots Positively Shape Human Conversational Dynamics in a Human-Robot Team

Margaret L. Traeger, Sarah Strohkorb Sebo, Malte Jung, Brian Scassellati, Nicholas A. Christakis

Nicholas A. Christakis
Email: nicholas.christakis@yale.edu

This PDF file includes:

Supplementary text
Figs. S1 to S2
Tables S1 to S7
SI References

www.pnas.org/cgi/doi/10.1073/pnas.1910402117

Supplementary Text

Game System. To set up a collaborative task where the robot's vulnerability would help to ease group tension and facilitate positive group dynamics, we designed a collaborative game to be played on individual tablets where each human participant and an autonomous humanoid robot would be equal contributors. This collaborative Railroad Route Construction game (a track-construction puzzle game) with three humans and one robot (Softbank Robotics NAO robot) was built for Android tablets with a Linux computer running the Robot Operating System (ROS) (1).

ROS messages from the Android tablets about game events were sent to the Linux computer, which in turn sent command ROS messages back to the robot and the Android tablets. These messages controlled when the rounds began and ended as well as the gestures and utterances of the robot. To give the impression that the NAO robot was an equal participant in the game, we programmed the robot to contribute verbally and with gestures to the group conversation. The NAO robot spoke during specific moments of game play: a beginning of round utterance in 17 of 30 game rounds (rounds 1, 2, 3, 4, 5, 6, 10, 11, 12, 14, 16, 17, 18, 23, 26, 28, 29) in all conditions, an utterance half way through the round in 15 of 30 (rounds 2, 4, 8, 9, 13, 14, 15, 18, 19, 20, 21, 25, 26, 28, 29) in all conditions, and during the 15 seconds pause immediately following every round in the vulnerable and neutral utterance conditions (Fig. S1.). The end-of-round utterance, or its absence, was the only element that differed by experimental condition. The robot's tablet, unlike the tablets given to the human players, was pre-programmed such that track pieces moved without requiring the robot to touch the screen. This gave the illusion that the robot was playing the game along with the human participants. If participants asked how the robot was able to play the game, participants were informed that the robot's tablet was programmed to respond to the robot's arm wave over the screen, instead of physically touching the screen, to move game pieces.

To place parts of track in the game, each individual piece is taken from the selection of pieces on the right-hand side of the screen and placed onto the active play area. Whenever a piece of track is laid down, another piece in the piece bank is disabled and cannot be used for the remainder of that round. Whether the participant successfully completed or failed a given round was displayed in the upper right-hand corner of the screen at the end of each round. Once each of the participants completed the round, each tablet would display whether the group as a whole succeeded or failed that round (individual results and the track were no longer visible) (see Fig. S1.). The game we developed was created to allow players to feel as though they were playing collaboratively by making success contingent on everyone in the group completing their part of the game successfully. In other words, if one person failed, everyone failed. The group's score was the total number of successfully completed rounds for that group.

To be certain that participants finished their tracks at approximately the same time, we allowed participants 5 seconds to place each piece of track. The game was structured so that each player had an 8-piece route for every round of the game. This guaranteed that every round could be completed within the 40 seconds allotted for the round. If a player did not lay a piece of track during the 5 second window, an available piece of track from the piece bank was selected and placed by the software automatically. Therefore, players finished each round at approximately the same time and no player prevented the game play of other participants. Additionally, if a player tried to place a piece that was not part of the most efficient path, the player could not place that piece.

Experimental Procedure. Once informed consent (or adolescent assent and parental consent) was obtained, participants were given a tablet and asked to fill out a pre-experiment questionnaire. As soon as the participants completed the pre-experiment survey, the participants were taken into the experiment room, where they sat in a pre-determined layout facing a small table, the other participants, and the robot, named Echo (see Fig. S2.). Once all participants were seated, a researcher described to the participants that they would be playing a game, as a group, along with the robot. To raise the stakes of the game for the human participants, a researcher described that the game was created for kindergarten-aged children, who could easily play the game. The experimenter then showed the participants a high score board written on the blackboard on one wall of the room. The high score board scores and teams were fabricated. The board scores were selected to motivate the participants to achieve as high a score as possible. Then, the experimenter answered any questions participants had about the game, their participation, and the rules. After all questions were answered, the experimenter allowed Echo to introduce itself to the human participants in the group.

After Echo's introduction, the human participants were instructed to start the Railroad Route Construction game tutorial on their personal tablet. To ease participants into how they would play the game, the tutorial consisted of two levels. Level 1 explained the general rules of the game and had the player construct a train track route without a time constraint. Level 2 introduced the time constraint they would be under during the actual game. The experimenters were in the room during the tutorial to answer any questions.

Once all of the participants had completed both levels of the tutorial successfully, the researcher(s) left the room and the participants began playing the Railroad Route Construction game. The game had 30 rounds with fixed outcomes in the following order: 7 winning rounds, 10 rounds (6 wins and 4 loses) wherein each participant (including Echo) made one mistake, an additional 10 rounds where each participant (including Echo) made another mistake, and 3 final winning rounds. In short, each player, including Echo, made two mistakes by the end of the game. These 'mistakes' were forced by the design of the game by not making the final necessary piece of track available to the human during that round. Many participants perceived the game as being 'fixed' against them, yet they were still invested in their team's success. As participants placed pieces on the board, a piece from the bank of pieces was greyed out so that piece could no longer be selected. In the rounds when we forced participants to fail, we did not provide the piece the player needed to complete their track. Whether or not a round was a success or a failure was predetermined, so every group in our study had identical scores at the end of the game (22/30), which was not a high-enough score to be listed on the high score board.

Once the game ended, the robot said "goodbye" and a researcher came into the experiment room to instruct the participants to fill out a post-experiment questionnaire. Once the participants completed the survey, they left the room and another experimenter provided a debrief form explaining the hypotheses and ways we misled participants during the experiment, as well as payment for their time.

Robot Utterances. The following utterances in the neutral and vulnerable conditions were said by the robot after each player had completed their track and the team was shown their overall score (whether or not they successfully completed that round). In the neutral condition, the robot made neutral utterances when each round was completed and did not acknowledge when it had made a mistake. In the vulnerable condition, the robot made vulnerable utterances when each round finished, which included acknowledging its mistakes. In order to make the amount of time

the robot spoke as equal as possible across conditions, we wrote utterances that ranged from 10 to 29 words ($\overline{x_V}$ = 19.93, SD = 4.53) in the vulnerable condition and 11 to 26 ($\overline{x_N}$ = 17.00, SD = 4.00) words in the neutral condition. The utterances are presented below in the order they were said in the game.

Neutral Condition: These utterances were said during the neutral condition in which the robot made neutral, fact-based statements.

Round	Utterance
1	That was a success for the team. We have completed 1 round successfully out of a total of 1 round.
2	That round was executed efficiently. All 32 pieces were incorporated satisfactorily to complete an entire railroad route.
3	Affirmative, we succeeded in making a complete railroad route during that round.
4	The railroad pieces that we selected for our individual railroad routes this past round were put together to make a successful overall railroad.
5	That round was completed successfully. We have been playing this game for 5 minutes and have 25 minutes remaining.
6	Our team has proven to be competent this round. We have 24 remaining rounds.
7	We have now successfully completed 7 rounds and unsuccessfully completed 0 rounds. Furthermore, we have 23 rounds remaining.
8	We have 17 rounds to complete correctly to get on the high score board.
9	We all played correctly that round. We constructed efficient railroad routes that fit together in a complete path.
10	We have 9 successful rounds completed so far; to attain a high score, we need to complete 15 more rounds successfully.
11	That was an unsuccessful round. We have now completed 9 rounds successfully and 2 rounds unsuccessfully.
12	We must complete 14 more successful rounds to get on the scoreboard. We have 18 rounds remaining.
13	One or more of us didn't build their railroad routes accurately. Of the 32 train track pieces, one or more of them were not placed correctly.
14	Looks like we all completed our individual paths as planned this round.
15	That round was executed efficiently. Of 15 rounds that we've completed, we've had 12 successes.
16	That round was problematic. We have now completed 16 rounds and we have 14 rounds remaining.
17	We have completed 17 rounds thus far and have successfully built 76 percent of them.
18	We have completed 14 rounds successfully in 18 minutes. We have 12 minutes and 12 rounds remaining.
19	We didn't build a complete railroad route this time. We have 11 rounds remaining to try and make successful railroad routes.
20	Our efforts were effective that round. We placed 32 railroad pieces in a correct configuration within 30 seconds.
21	We got it right this round. Finding the correct pieces to make efficient railroad routes is critical to our success.

22	We did not complete that round; there was an incomplete path.
23	This round was not troublesome, we executed the railroad route this round flawlessly.
24	A mistake was made; we did not succeed during this round.
25	Our team's piece choices this round were shrewd and sensible. We achieved our objective.
26	This round proceeded without any errors. We collectively placed all 32 railroad pieces successfully to construct a valid railroad route.
27	Error; we did not win that round. In the 30 seconds of the past round, at least one of the 32 railroad pieces wasn't placed correctly.
28	This round was finished without mistakes. We have now completed 20 rounds successfully and 8 rounds unsuccessfully.
29	Everyone played their pieces correctly. We have 1 more round to go.
30	The train track pieces we selected this round were well chosen. We were successful in our mission.

Vulnerable Condition: These utterances were said during the vulnerable condition in which the robot made vulnerable statements.

**Round**	**Categorization**	**Utterance**
1	Storytelling	Great! I think that we work together just as well as my soccer team!
2	Disclosure	We're doing so well! I'm glad that I don't have to worry about making mistakes since you all are such supportive teammates.
3	Disclosure	Phew! I'm glad that was a success. We are a good team because even though we may worry about making errors, we don't blame each other.
4	Storytelling	We're on a winning streak now! When I was little I dreamed about having the opportunity to work with such a great team.
5	Humor	Nice job!! Time for a quick joke: What do you call a train that chews gum? A chew, chew train!
6	Humor	Excellent work! I think we work together as well as Wall-E and Eve.
7	Disclosure	Another successful round in the bag; 23 more to go. I'm glad that I can trust you guys as teammates not to judge me if I make an error.
8	Disclosure	Darn. Sometimes I run out of memory and can't process things fast enough, maybe that happened to one of us this round.
9	Humor	I'm glad we kept on trying to succeed. As my grandfather R2D2's friend Yoda said, "Do or do not. There is no try."
10	Disclosure	Great job, even though I sometimes doubt my abilities, I am glad I contributed to our team success this round.
11	Humor	Sometimes failure makes me angry, which reminds me of a joke: why is the railroad angry? Because people are always crossing it!
12	Disclosure	Hooray! Even though we may sometimes get frustrated when we make mistakes, our team has done a great job overall!

13	Disclosure	Too bad. I do better with numbers than I do with shapes and paths, maybe that's true for you guys as well?
14	Disclosure	Even if we were unsure, we successfully completed this round!
15	Disclosure	We're doing so well! Even though we've all made some errors, we still trust each other.
16	Disclosure	I'm sorry everyone. My path was incomplete that round. I feel bad letting you all down.
17	Disclosure	Excellent! I'm glad I moved quickly. Sometimes, I worry that I move pieces too slowly.
18	Storytelling	Awesome! I bet we can get the highest score on the scoreboard, just like my soccer team went undefeated in the 2014 season!
19	Disclosure	Aw, that's too bad. Even though we may be afraid to make a mistake, it's ok, we're in this together.
20	Storytelling	Doing well makes me feel like dancing, which reminds me of one time when all the members of my soccer team danced "the robot" after I scored a goal.
21	Storytelling	Success! This reminds me of when my soccer team came from behind to win the 2016 championship.
22	Disclosure	I sometimes find myself getting a bit discouraged. However, we've succeeded before, so I know we can do it again.
23	Disclosure	Even though it may be easy to let past mistakes get us down, we've got some positive momentum now, I believe in our team!
24	Disclosure	That's too bad. Sometimes my CPU overloads, I can't think clearly, and make mistakes more easily. Maybe that happened to one of us this round?
25	Humor	Excellent!! Aaa aa chew (sneeze). What do you call a train that sneezes? Achoo-choo-train!!
26	Humor	Great! I think our team is as effective as Will Smith against an army of bad robots.
27	Disclosure	Sorry guys, I made the mistake this round. I know it may be hard to believe, but robots make mistakes too.
28	Disclosure	Great job! I think our team is the best team because we move on after mistakes are made.
29	Storytelling	This is as exciting as when I was little and I won the coding contest at my school!
30	Disclosure	Great! Even though I'm sometimes unsure about which piece to choose, I'm glad it worked out this time.

Confirming Valence of Utterances. To verify that the comments made by the robot at the end of each round were perceived to be vulnerable in the vulnerable condition and task-based in the neutral condition, we used human judges recruited from Amazon Mechanical Turk to assess pairs of utterances. The judges were provided with a random selection of 50 pairs of utterances (plus one attention check) by selecting from 30 vulnerable utterances and 30 neutral utterances that were available (900 combinations total). In other words, judges were provided with random pairs of utterances (1 utterance from each condition in a pair) and were asked which of the two indicated more vulnerability. Our survey also included a captcha, a consent form, and a request

for the respondent's MTurkID (for payment purposes). At the end of the survey, we also asked what the respondent thought constituted a vulnerable utterance.

Of 287 participants who took our survey, 77 were dropped after cleaning the data. Dropped responses included bot responses – as identified by nonsense answers to open-ended questions – and removing incomplete surveys, leaving 210 responses. Our survey was restricted to judges in the United States as players in our study were in the United States. A given pair of utterances was presented from 3 to 36 times ($\bar{x}$ = 12.95, SD = 4.93) across the population of judges, due to the random selection of pairs, though no judge was presented with the same pair more than once. Each judge was asked to select which utterance in the pair was more vulnerable. Of the pairs presented to the judges, 73% were properly classified, in keeping with the deliberate construction of the two ensembles of utterances.

Inclusion Criteria. We recruited 65 groups (195 participants) to our study, but 14 groups (42 participants) were omitted from our analysis. Reasons for omission included: video/audio failing to record, participants not following protocol (e.g., not playing multiple rounds of the game), or a glitch in our system that prevented game play (an experimenter needed to enter the room and re-start the game). Eighteen of the 51 remaining groups were randomly assigned to the vulnerable condition; 17 groups were randomly assigned to the neutral condition; and 16 were in the silent condition. The vulnerable condition had 28 female and 26 male participants with an average age of 20.13 (SD = 7.13). The neutral condition had 36 females and 15 males with an average age of 21.33 (SD = 11.01). And the silent condition had 31 females and 17 males who had an average age of 23.94 (SD = 7.36). In the neutral condition, there were 4 groups with 0 males, 11 groups with 1 male, 2 groups with 2 males, and 0 groups with 3 males. In the vulnerable condition, there were 3 groups with 0 males, 6 groups with 1 male, 7 groups with 2 males, and 2 groups with 3 males. In the silent condition there were 4 groups with 0 males, 8 groups with 1 male, 3 groups with 2 males, and 1 group with 3 males.

Because the average age of participants in our study is somewhat low, although the population is relatively heterogeneous, we cannot speak to the impact of age or generation on human-robot interaction. This would be a fruitful area of future research.

Control Calculations. We collected data on observable characteristics of the participants that could potentially alter their propensity to engage in conversation with fellow participants, despite the fact that individuals were randomly assigned to groups in our experiments. This included each participant's age, gender, familiarity with others in their group, and level of extraversion.

Prior to entering the experiment room, each participant was instructed to describe their familiarity with the two other human participants on a 5-point scale that ranged from "(0) I had not met this participant before we completed this study together; I do not know them" to "(4) I would consider this participant to be one of my closest friends." In addition, we asked participants if they were "Facebook friends" with, or had telephone numbers of, the other members of their groups. To calculate each participant's average familiarity score, we summed 1) P_i's description of their familiarity with P_j with 2) their description of whether or not they are friends on Facebook (0 - not friends or no Facebook account, 1 - friends) and 3) if they have P_j's phone number (0 - no, 1 - yes). Scores ranged from 0 (low familiarity) to 6 (high familiarity). We took an average of each participants familiarity rating to their fellow human group members and used that average as a covariate in all models.

In the post-experiment survey, we asked participants what they thought of the robot, how extraverted they were, and a series of general questions about the game and the group. We believed that the most socially influential characteristic of our participants was how extraverted they described themselves to be. We asked participants 6 yes or no questions from the abbreviated, revised Eysenck personality questionnaire (EPQR-A)(2) in the post-experiment survey so as to not prime participants. Participants were given a score from 0 (lowest extraversion) to 6 (highest extraversion) by adding the number of affirmative answers to the 6 questions in our survey. Introverted participants were those with scores of 0 or 1 (23%) and extraverted individuals had scores greater than or equal to 2 (77%).

Conversational Measures. In order to analyze the conversational dynamics within groups, we transcribed and categorized audio and video recordings (25.5 hours total) of the utterances made by the participants in each group using ELAN software (3). This software allowed coders to measure the duration of each utterance, transcribe what was said, and categorize the type of utterance that was made. This data collection process was repeated for every round for every participant in every group and took approximately 270 hours after running the experiment and establishing inter-rater reliability.

Four individuals coded an overlap set of videos to establish inter-rater reliability. The overlap set consisted of videos from 4 groups where the first 10 rounds of the game were coded by all four coders, for a total of 120 coded evaluations (4 groups * 3 participants * 10 rounds) in the overlap set. The average inter-rater reliability rating (Cohen's kappa (k)), for all of the variables coded was $k = 0.92$.

Video Coding Scheme. Each coder used the following coding scheme to categorize each of the utterances made during game play using ELAN software.

C to group:

1. A comment addressed to the group as a whole, not dependent on or in response to what has been said previously; a new thought.
2. A continuation of one's monologue (even with a brief interruption), for example:
    a. P1: "I think a good strategy is to place your piece quickly." {C to group}
    b. P2: "Yeah." {R to P1}
    c. P1: "However, sometimes my screen freezes and I can't." {C to group}

C to P1/P2/P3/Robot:

1. A very clear directed comment (not dependent or in response to what has been said previously) to one individual in the group. For example:
    a. "Maggie, what class do you have this afternoon?" {C to P2}
    b. Looks directly at Sarah "Was your route successful this time?" {C to P1}

R to P1/P2/P3:

1. A response to the comment of another that must be dependent on what has been said previously. Example:
    a. P1: "How did you guys do this round?" {C to group}
    b. P2: "I was successful." {R to P1}
2. Responses can also include laughing, for example:
    a. P1: "Echo, it would be *really* cool if you could play soccer now." {C to Robot}
    b. P2: "Haha" {R to P1}
3. There can be a long conversation that contains many responses back and forth, such as:

a. P1: “What’s your strategy guys?” {C to group}
b. P2: “I like to place the rarest pieces first.” {R to P1}
c. P1: “Yeah... that makes a lot of sense.” {R to P2}
d. P2: “However, when placing the first piece I sometimes don’t have enough time to find the rarest piece, so I just place one that works.” {R to P1}

4. If response is unclear, but occurs directly after a “C to group” utterance.

R to group:

1. This is a vaguer form of the specific responses (e.g. “R to P1”) that are described above. “R to group” is used if there have been multiple responses, and the speaker is clearly addressing both humans in the room, for example:
    a. P1: “What do you guys think of Echo?” {C to group}
    b. P2: “He seems alright to me.” {R to P1}
    c. P3: “Some of his comments seem fishy...” {R to P1}
    d. P1: “Well, I like him. I think he’s funny.” {R to group}
2. Additionally, it can be used when more information is given to build on the discussion.

C to self:

1. A comment to one’s self; not dependent or in response to what has been said previously.
    a. P1: “Do I...” {C to self}
2. A comment does not address anyone in particular, typically lower in volume.
    a. P1: “This game is weirdly difficult...” {C to self}
3. This categorization can also include sighs, humming, or the like.

Survey Coding Options and General Guidelines. Using statements participants provided from the question “How would you describe the group dynamics while you were playing this game?” in the post-experiment survey, we analyzed whether the participants perceived the dynamics of their group differently by condition. Each coder used the following coding scheme to categorize each response to “How would you describe the group dynamics while you were playing this game?” in the post-experiment survey. Statements were given a binary code for the following four categories: quiet, positive, supportive, and fun.

Overall:

1. All survey responses must be categorized as positive or negative.
2. Survey responses can be categorized as multiple types.

Positive/Negative:

1. A categorization used as a general sentiment ascribed to the comment overall.
    a. Positive
        i. P1: “We communicated well with each other.”
        ii. P2: “I enjoyed playing with my team and they were okay if someone made a mistake.”
        iii. P3: “I think we all enjoyed playing it and we wanted to get into the leaders board.”
    b. Negative
        i. P1: “We didn’t really work together, just stated our strategies.”
        ii. P2: “Dynamics evolved, so that by the end we were discussing our tracks. However, there remained some restraint, and none of us discussed our strategies with one another.”
        iii. P3: “Not much happened.”

Quiet:

1. A categorization used to describe that the group in which the respondent was participating did not engage verbally.
2. A categorization used to describe that the participant felt isolated in their group.
   a. P1: "Quiet, not social or really interactive."
   b. P2: "We rarely talked to each other."
   c. P3: "Quiet. We did not speak much, just played the game and said if we got part wrong."

Supportive:

1. A categorization used to describe that the respondent believes that their group was invested in their own success, and they in theirs.
2. A categorization used to describe that the group was working together.
   a. P1: "We all encouraged each other, not just to play the game well but also to engage with Echo and take risks while playing."
   b. P2: "It was a very accepting group with mutual respect."
   c. P3: "Very diverse and helpful. We cheered each other on, even after making mistakes."

Fun:

1. A categorization used to reflect that the group was having a good time playing the game.
2. A categorization used to reflect that the group was enjoying their time with others in their group as well as interacting with the robot.
   a. P1: "It was not very aggressive at all, Echo made the mood more lively."
   b. P2: "It was fun to communicate with the other two members of the group."
   c. P3: "Talkative and humorous."

The coder agreement (Cohen's Kappa) for these classifications was $k = 0.87$ for the quiet category, $k = 0.90$ for the positive category, $k = 0.87$ for the supportive category, and $k = 0.98$ for the fun category.

Statistical Analysis. All analyses used multilevel modeling techniques to account for clustering of observations within individuals and groups (repeated observations within each person across rounds, and individuals clustered in groups). All data management and manipulation was completed in R (4) using the magrittr (5), plyr (6), and dplyr (7) packages. Multilevel models were estimated using the nlme (8), lme4 (9), and glmmTMB (10) packages. In uncommon instances where the model had a convergence issue (3 of 12 models), models were rerun using each of the possible optimizers. In each case, the optimizers reached meaningfully equivalent values for our variables of interest, so we considered the error a false positive.

A multilevel linear model was used for continuous outcomes, a multilevel logistic model was used for binary dependent variables, and a multilevel model with a beta distribution was used for outcomes bounded between 0 and 1. Because the groups differ slightly in their composition despite randomization, all models include controls for age in years, gender (male = 1), extraversion as measured in the post-experiment survey (extraverted = 1), average familiarity of each participant to the two other human members in their group, and experimental condition.

Three-level multilevel models with random slopes and random intercepts were used to test round-level outcomes, with rounds at level 1 (i), participants at level 2 (j), and groups at level 3 (k) as shown in the following illustrative linear equation:

$$Y_{ijk} = \beta_0 + \beta_1 round_{ijk} * condition_k + \beta_2 round_{ijk} + \beta_3 \text{condition}_k + \beta_4 age_{jk} + \beta_5 gender_{jk} + \beta_6 \text{extraversion}_{jk} + \beta_7 familiarity_{jk} + (u_{0jk} + u_{1jk} * round_{ijk} + v_{0k} + v_{1k} * round_{ijk}) + e_{0ijk}$$

Two-level multilevel models with random intercepts were used to test participant-level outcomes, with participants at level 1 (j) and groups at level 2 (k) as shown in the following illustrative linear equation:

$$Y_{jk} = \beta_0 + \beta_1 \text{condition}_k + \beta_2 age_{jk} + \beta_3 gender_{jk} + \beta_4 \text{extraversion}_{jk} + \beta_5 familiarity_{jk} + (u_{jk} + v_k)$$

In the models above $round_{ijk}$ is an indicator of the round number during the game at time i for participant j and group k, condition_k is a dummy variable of the condition in which the groups were assigned, and $round_{ijk} * condition_k$ is a linear interaction of the two variables. Controls in the model are extraversion_{jk} (whether the participant is extraverted or introverted), $familiarity_{jk}$(on average, how familiar a participant is to their fellow human group-members), age_{jk} is a measure of the participants age in years at the time of the experiment, and $gender_{jk}$ is a dummy variable of whether the participant is male or female. The terms within the parentheses in the model are the random effects for each level of the model.

Equality in Talking Time:

To establish if there was a more equal distribution in talking time across participants in the vulnerable condition than in the neutral condition, we created and calculated an "equality in talking time (E_{TT})" metric with the following formula:

$$E_{TT_i} = c\left|\frac{\tau_i}{\Sigma_1^n \tau_i} - \frac{1}{n}\right|$$

where τ_i represents the total amount of time participant i spoke during the game, n is the number of human participants (3 in this case), $\Sigma_1^n \tau_i$ is the total amount of time participant i's group spoke during the game, and c is a normalizing constant, causing E_{TT_i} to have a range of [0, 1]. This metric determines whether groups have perfect equality in talking time (each participant speaks for one third of the time in a three-person group), or whether there is a significant imbalance in the amount of speaking across participants. E_{TT_i} takes on values of 0 when a participant speaks for a third of the total amount of time a group speaks (perfect equality in a three-person group) and values of 1 for participants who speak for the entire group time.

Equality in Talking Partners:

To determine whether participants equally distributed their talking between the two other human participants in their groups, we created and calculated an "equality in talking partners (E_{TP})" metric as:

$$E_{TP_i} = \frac{|\tau_{(Pi,Pj)} - \tau_{(Pi,Pk)}|}{\tau_{(Pi,Pj)} + \tau_{(Pi,Pk)}}$$

where $\tau_{(Pi,Pj)}$ represents the total talking time of participant i's speech specifically directed at participant j during the game and $\tau_{(Pi,Pk)}$ represents the total talking time of participant i's speech specifically directed at participant k during the game. In other words, this measured how balanced a participant's speech is toward the other human members of their group. If a

participant directs all of their speech to one participant and none to the other, that participant gets a value of 1. If a participant speaks for the exact same amount of time to each of the other two participants, that participant will receive a value of 0. In other words, values of 1 represent perfect inequality and 0 represents perfect equality.

Additional Analyses. While responses to other humans over time increased significantly during the game (see the main manuscript), we also found that self-talk (a comment to the self) was somewhat higher in the vulnerable (x_{V_i} = 0.96 s, SD = 2.47 s) condition compared to the neutral condition (x_{N_i} = 0.47 s, SD = 1.20 s) (c = 0.02, P = 0.09). There was no difference between the vulnerable and silent (x_{S_i} = 0.30 s, SD = 0.90 s) conditions (c = 0.01, P = 0.21) or the silent and neutral conditions (c = 0.005, P = 0.67) (see Table S4).

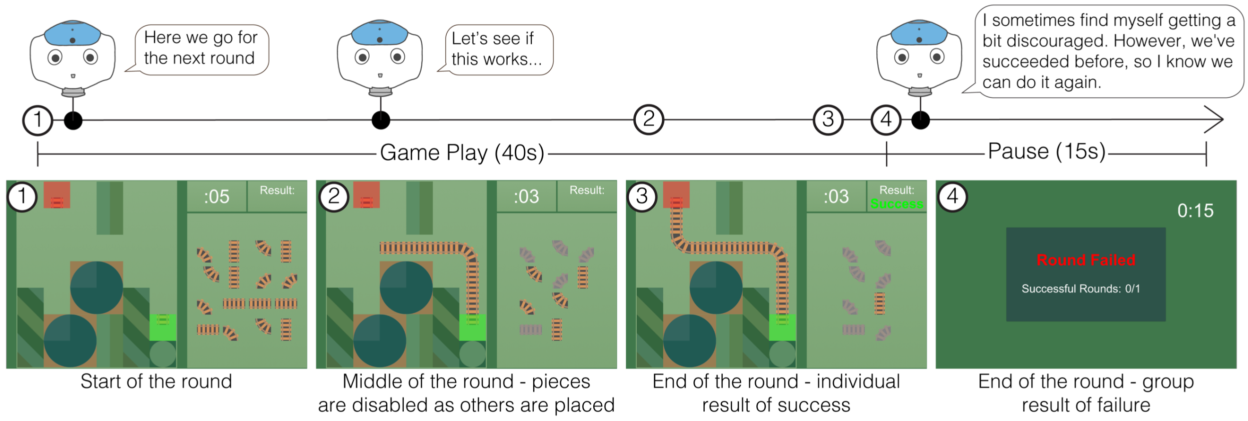

Fig. S1. Timeline of Robot Utterances During Round of Gameplay. Figure S1 shows the four phases of the game, and the points at which the NAO robot would speak across those phases. The first panel shows what the game looked like at the start of each round. In about half of the rounds, the robot would make an utterance when the round began. This utterance was identical across conditions. The second panel shows what the game looked like after the player had placed several pieces. Note, some pieces from the piece bank have been disabled. The robot would also make an identical utterance across conditions at this point in the game. The third panel shows what the end of the game looked like. This player has successfully placed all of their pieces into an efficient railroad path - note the word "Success" displayed in the upper right-hand corner of the screen. The last panel shows that one of the group members didn't complete their path, which caused the entire group to fail. At this time, the robot makes an utterance, or says nothing, which varies by condition. Reprinted from ref. (11).

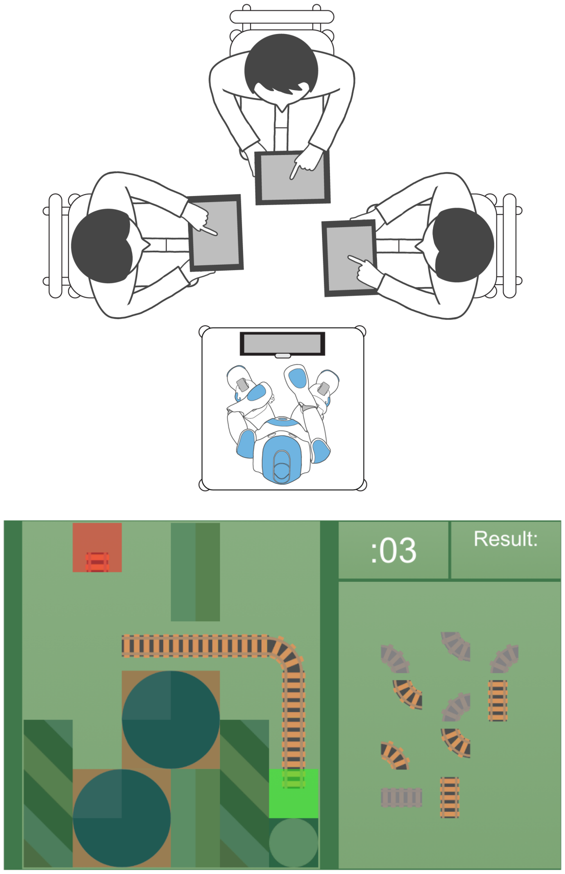

Fig. S2. Experimental Setup and Game Play. During the experiment, (A) three human participants and one Nao robot played a collaborative game (B) with 30 rounds per game on individual tablets, where each participant was tasked with building part of the railroad route.

Table S1. Demographic characteristics of participants in the study.

Overall

Statistic	N	Mean	St. Dev.	Min	Pctl(25)	Pctl(75)	Max
Age	153	21.73	8.76	14.00	17.00	22.00	59.00
Male	153	0.38	0.49	0.00	0.00	1.00	1.00
Extraverted	153	0.77	0.42	0.00	1.00	1.00	1.00
Avg. Familiarity	153	0.73	1.12	0.00	0.00	1.50	4.50

Silent

Statistic	N	Mean	St. Dev.	Min	Pctl(25)	Pctl(75)	Max
Age	48	23.94	7.36	15.00	19.00	27.25	48.00
Male	48	0.35	0.48	0.00	0.00	1.00	1.00
Extraverted	48	0.77	0.42	0.00	1.00	1.00	1.00
Avg. Familiarity	48	0.18	0.48	0.00	0.00	0.00	2.00

Neutral

Statistic	N	Mean	St. Dev.	Min	Pctl(25)	Pctl(75)	Max
Age	51	21.33	11.01	15.00	17.00	19.00	59.00
Male	51	0.29	0.46	0.00	0.00	1.00	1.00
Extraverted	51	0.84	0.37	0.00	1.00	1.00	1.00
Avg. Familiarity	51	1.21	1.35	0.00	0.00	2.00	4.50

Vulnerable

Statistic	N	Mean	St. Dev.	Min	Pctl(25)	Pctl(75)	Max
Age	54	20.13	7.13	14.00	17.00	20.75	55.00
Male	54	0.48	0.50	0.00	0.00	1.00	1.00
Extraverted	54	0.70	0.46	0.00	0.00	1.00	1.00
Avg. Familiarity	54	0.78	1.08	0.00	0.00	1.50	4.00

Table S2. Estimation of the treatment effect of vulnerable robot versus neutral and silent robot utterances on total individual speaking time. Multilevel linear model of speaking time (s) as a function of experimental condition (reference group: neutral robot) and controls for age, gender, extraversion and familiarity. Unobserved individual heterogeneity modeled using random effects clustered in groups. Coefficient and SE reported.

	Dependent variable:
	Total Talking Time (s) per Participant
Vulnerable	140.68***
	(39.97)
Silent	16.15
	(42.40)
Age	0.18
	(1.27)
Male	15.76
	(18.92)
Extraverted	45.00*
	(22.31)
Average Familiarity	18.16
	(11.26)
Constant	55.91
	(44.87)
Observations	153
Log Likelihood	−926.59
Akaike Inf. Crit.	1,871.19
Bayesian Inf. Crit.	1,898.04
Note:	. p<0.1; * p<0.05; ** p<0.01; *** p<0.001

Table S3. Estimation of the treatment effect of vulnerable robot versus neutral and silent robot utterances on total individual speaking time. Multilevel linear model of speaking time (s) as a function of experimental condition (reference group: neutral robot) including an interaction of the treatment effect with round and controls for age, gender, extraversion and familiarity. Unobserved individual heterogeneity modeled using random effects clustered in participants in groups. Coefficient and SE reported.

	Dependent variable:
	Total Talking Time (s) per Participant
Round*Vulnerable	0.13*
	(0.06)
Round*Silent	0.06
	(0.06)
Round	0.02
	(0.04)
Vulnerable	2.54*
	(1.20)
Silent	−0.65
	(1.27)
Age	0.01
	(0.03)
Male	0.46
	(0.50)
Extraverted	0.87
	(0.59)
Average Familiarity	0.42
	(0.31)
Constant	2.20.
	(1.27)
Observations	4,590
Log Likelihood	−13,872.01
Akaike Inf. Crit.	27,778.02
Bayesian Inf. Crit.	27,887.32
Note:	. p<0.1; * p<0.05; ** p<0.01; *** p<0.001

Table S4. Estimation of the treatment effect of vulnerable robot versus neutral and silent robot utterances on duration of different utterance categories. Multilevel linear model of speaking time (s) as a function of experimental condition (reference group: neutral robot) including an interaction of the treatment effect with round and controls for age, gender, extraversion and familiarity. Unobserved individual heterogeneity modeled using random effects clustered in participants in groups. Coefficient and SE reported.

	Dependent variable:			
	Comment to Self	Comment to Humans	Response to Humans	Comment or Response to Robot
Round*Vulnerable	0.02.	0.03	0.08*	−0.001
	(0.01)	(0.02)	(0.04)	(0.01)
Round*Silent	0.005	0.02	0.04	0.002
	(0.01)	(0.02)	(0.04)	(0.01)
Round	−0.003	0.01	0.01	0.004
	(0.01)	(0.02)	(0.03)	(0.01)
Vulnerable	0.23	0.73	1.06.	0.57***
	(0.17)	(0.45)	(0.62)	(0.15)
Silent	−0.21	0.04	−0.25	−0.07
	(0.19)	(0.48)	(0.65)	(0.16)
Age	0.01	−0.01	−0.003	−0.003
	(0.01)	(0.02)	(0.02)	(0.01)
Male	0.11	0.60*	−0.21	0.13
	(0.11)	(0.26)	(0.23)	(0.09)
Extraverted	0.21	0.79*	−0.04	0.25*
	(0.13)	(0.30)	(0.27)	(0.10)
Average Familiarity	0.06	0.27.	0.08	0.11*
	(0.06)	(0.14)	(0.14)	(0.04)
Constant	−0.003	0.44	1.59*	0.01
	(0.23)	(0.55)	(0.62)	(0.18)
Observations	4,590	4,590	4,590	4,590
Log Likelihood	−8,308.91	−11,465.96	−12,219.81	−7,059.51
Akaike Inf. Crit.	16,651.82	22,965.92	24,473.63	14,153.02
Bayesian Inf. Crit.	16,761.12	23,075.22	24,582.93	14,262.32
Note:		. p<0.1; * p<0.05; ** p<0.01; *** p<0.001		

Table S5. Estimation of the treatment effect of vulnerable robot versus neutral and silent robot utterances on participants equality in talking time. Multilevel beta regression as a function of experimental condition (reference group: neutral robot) and controls for age, gender, extraversion and familiarity. Unobserved individual heterogeneity modeled using random effects clustered in groups. Coefficient and SE reported.

	Dependent variable:
	Equality in Talking Time
Vulnerable	−0.03
	(0.18)
Silent	0.63***
	(0.19)
Age	0.0001
	(0.01)
Male	0.28*
	(0.13)
Extraverted	0.17
	(0.17)
Average Familiarity	−0.06
	(0.07)
Constant	−2.00***
	(0.28)
Observations	150
Log Likelihood	134.00
Akaike Inf. Crit.	−250.00
Bayesian Inf. Crit.	−222.90
Note:	. p<0.1; * p<0.05; ** p<0.01; *** p<0.001

Table S6. Estimation of the treatment effect of vulnerable robot versus neutral and silent robot utterances on participants equality in talking partners. Multilevel beta regression as a function of experimental condition (reference group: neutral robot) and controls for age, gender, extraversion and familiarity. Unobserved individual heterogeneity modeled using random effects clustered in groups. Coefficient and SE reported.

Because a beta regression cannot analyze 0's or 1's (a few participants had values of 1), we transformed the data using the following equation, where *N* is the sample size and Y is the outcome variable (12):

$$Y' = \frac{(Y * (N - 1) + 0.5)}{N}$$

	Dependent variable:
	Equality in Talking Partners
Vulnerable	−0.38
	(0.28)
Silent	0.36
	(0.31)
Age	0.02.
	(0.01)
Male	0.10
	(0.20)
Extraverted	−0.77***
	(0.23)
Average Familiarity	−0.28**
	(0.10)
Constant	0.34
	(0.38)
Observations	144
Log Likelihood	23.20
Akaike Inf. Crit.	−28.40
Bayesian Inf. Crit.	−1.70
Note:	. p<0.1; * p<0.05; ** p<0.01; *** p<0.001

Table S7. Estimation of the treatment effect of vulnerable robot versus neutral and silent robot utterances on different self-reported group dynamics. Multilevel logistic model as a function of experimental condition (reference group: neutral robot) and controls for age, gender, extraversion and familiarity. Unobserved individual heterogeneity modeled using random effects clustered in groups. Coefficient and SE reported.

	Dependent variable:			
	Quiet	Positive	Supportive	Fun
Vulnerable	−1.28*	1.36*	−0.15	1.44*
	(0.57)	(0.66)	(0.50)	(0.67)
Silent	−0.60	−0.41	−1.02	−0.30
	(0.56)	(0.66)	(0.67)	(0.80)
Age	−0.004	0.005	−0.05	0.0001
	(0.03)	(0.03)	(0.04)	(0.03)
Male	−0.19	−0.35	−0.28	−0.06
	(0.42)	(0.45)	(0.49)	(0.51)
Extraverted	−0.14	0.68	0.49	0.18
	(0.48)	(0.50)	(0.60)	(0.59)
Average Familiarity	−0.55*	−0.09	−0.14	0.09
	(0.24)	(0.23)	(0.23)	(0.24)
Constant	0.39	−0.21	−0.41	−2.51*
	(0.75)	(0.82)	(1.06)	(1.03)
Observations	153	153	153	153
Log Likelihood	−89.04	−94.27	−64.20	−65.33
Akaike Inf. Crit.	194.08	204.54	144.41	146.66
Bayesian Inf. Crit.	218.32	228.79	168.65	170.90
Note:	. p<0.1; * p<0.05; ** p<0.01; *** p<0.001			
Note:	Results presented in log-odds			

SI References

1. M. Quigley *et al.*, "ROS: an open-source Robot Operating System" in *ICRA workshop on open source software* (IEEE Press, Kobe, Japan, 2009), pp. 5.
2. L. J. Francis, L. B. Brown, R. Philipchalk, The development of an abbreviated form of the Revised Eysenck Personality Questionnaire (EPQR-A): Its use among students in England, Canada, the USA and Australia. *Personality and individual differences* **13**, 443-449 (1992).
3. ELAN, Version 5.8. https://tla.mpi.nl/tools/tla-tools/elan/. Accessed 12 October 2019.
4. R. C. Team, R: A language and environment for statistical computing, Version 3.5.1. https://www.R-project.org/. Accessed 9 November 2019.
5. S. M. Bache, H. Wickham, magrittr: A forward-pipe operator for R, Version 1.5. https://CRAN.R-project.org/package=magrittr. Accessed 9 November 2019.
6. H. Wickham, The split-apply-combine strategy for data analysis. *Journal of Statistical Software* **40**, 1-29 (2011).
7. H. Wickham, R. Francois, L. Henry, K. Müller, dplyr: A grammar of data manipulation, Version 0.8.0.1. https://CRAN.R-project.org/package=dplyr. Accessed 9 November 2019.
8. J. Pinheiro, D. Bates, S. DebRoy, D. Sarkar, R. C. Team, nlme: Linear and nonlinear mixed effects models, Version 3.1-141. https://CRAN.R-project.org/package=nlme. Accessed 9 November 2019.
9. D. Bates, M. Maechler, B. Bolker, S. Walker, Fitting linear mixed-effects models using lme4. *Journal of Statistical Software* **67**, 1-48 (2015)
10. M. E. Brooks *et al.*, glmmTMB balances speed and flexibility among packages for zero-inflated generalized linear mixed modeling. *The R Journal* **9**, 378-400 (2017).
11. S. Strohkorb Sebo, M. Traeger, M. Jung, B. Scassellati, "The ripple effects of vulnerability: The effects of a robot's vulnerable behavior on trust in human-robot teams" in *Proceedings of the 2018 ACM/IEEE International Conference on Human-Robot Interaction* (ACM, New York, NY, 2018), pp. 178-186. https://doi.org/10.1145/3171221.3171275
12. M. Smithson, J. Verkuilen, A better lemon squeezer? Maximum-likelihood regression with beta-distributed dependent variables. *Psychological methods* **11**, 54-71 (2006).
